# Tapioca Starch Improves the Quality of *Virgatus nemipterus* Surimi Gel by Enhancing Molecular Interaction in the Gel System

**DOI:** 10.3390/foods13010169

**Published:** 2024-01-03

**Authors:** Xiaobing Huang, Qingguan Liu, Pengkai Wang, Chunyong Song, Huanta Ma, Pengzhi Hong, Chunxia Zhou

**Affiliations:** 1 College of Food Science and Technology, Guangdong Ocean University, Zhanjiang 524088, China; huang14795223331@163.com (X.H.); liuqingguansdk@163.com (Q.L.); wangpengkai6688@163.com (P.W.); song_li2020@163.com (C.S.); 17876042414@163.com (H.M.); hongpengzhi@126.com (P.H.); 2 Guangdong Provincial Key Laboratory of Aquatic Product Processing and Safety, Zhanjiang 524088, China; 3 Guangdong Provincial Engineering Technology Research Center of Marine Food, Zhanjiang 524088, China; 4 Guangdong Modern Agricultural Science and Technology Innovation Center, Zhanjiang 524088, China; 5 Southern Marine Science and Engineering Guangdong Laboratory, Zhanjiang 524088, China

**Keywords:** surimi gel, tapioca starch, physicochemical properties, microstructure, molecular interaction

## Abstract

The gel prepared using *Nemipterus virgatus* (*N. virgatus*) surimi alone still has some defects in texture and taste. Complexing with polysaccharides is an efficient strategy to enhance its gel properties. The main objective of this study was to analyze the relationship between the gel quality and molecular interaction of *N. virgatus* surimi gel after complexing with tapioca starch. The results make clear that the gel strength, hardness, and chewiness of surimi gel were increased by molecular interaction with tapioca starch. At the appropriate addition amount (12%, *w*/*w*), the surimi gel had an excellent gel strength (17.48 N), water-holding capacity (WHC) (89.01%), lower cooking loss rate (CLR) (0.95%), and shortened *T*_2_ relaxation time. Microstructure analysis indicated that the addition of tapioca starch facilitated even distribution in the gel network structure, resulting in a significant reduction in cavity diameter, with the minimum diameter reduced to 20.33 μm. In addition, tapioca starch enhanced the hydrogen bonding and hydrophobic interaction in the gel system and promoted the transformation of α-helix to β-sheet (*p* < 0.05). Correlation analysis showed that the increased physicochemical properties of surimi gel were closely related to the enhanced noncovalent interactions. In conclusion, noncovalent complexation with tapioca starch is an efficient strategy to enhance the quality of surimi gel.

## 1. Introduction

*N. virgatus* is a low-value marine fish and the second-largest species for processing surimi products [1]. As is well known, the gel characteristics of surimi are a crucial indicator of its quality, which reduces during the production process. Therefore, a cheap and safe exogenous additive can be chosen to enhance the gel qualities of surimi products, which can help to efficiently utilize low-value fish and reduce production costs [2,3].

The starch is gelatinized by heating, and the internal particles are expanded to fill the network structure of the gel, thereby improving the quality of surimi products [4,5,6]. Different starches and dosages have different effects, and too much starch affects the quality of surimi [7,8]. Studies have found that when potato starch is added at 16% (*w*/*w*), the starch–protein interaction is the most conducive to promoting hairtail surimi gel [5]. However, others believe that adding 8% (*w*/*w*) of sweet potato starch has the best effect in improving the quality and network structure of surimi gel [9]. Therefore, there may be a saturation state for crosslinked protein to starches, which limits the texture properties of surimi gel. At the same time, some scholars have found that a different amount of starch added also makes a difference in changes to the chemical force in the surimi matrix [4,10], and the mechanisms that cause this change are different. It is believed that starch may interact with myofibrillar proteins through electrostatic or hydrogen bond interactions, contributing to improving the texture of surimi gels [11]. But it may also be that the destruction of starch structure after heating causes the macromolecular polymer to bind to myofibrillar protein residues, resulting in changes in chemical force [4]. This is because the enhancement in the texture properties of surimi gel and the change in intermolecular forces are affected by the type of starch and the amount of starch added. Therefore, it is still necessary to investigate the influence mechanism of different types and amounts of starch on the molecular structure and molecular interaction of myofibrillar protein and its relationship with the quality of surimi products.

Tapioca starch has a high amylopectin content and strong swelling ability, which causes surimi to form a strong adhesion gel [12]. Moreover, tapioca starch particles are larger, a slight expansion can produce a large viscosity, and the gel network structure has a good filling effect [13]. There are also some related studies on the effect of tapioca starch on the texture properties of surimi gel. It was found that the addition of tapioca starch and modified starch altered the physicochemical properties and protein structure of surimi gel in the low concentration range [8]. However, many studies have only compared the effects of natural tapioca starch with modified starch on the gel properties and microstructure of surimi [14,15]. A multiscale comprehensive analysis of natural tapioca starch and its added amount regarding the physicochemical properties, microstructure, and molecular interactions of surimi gel has not been investigated.

Tapioca starch with high amylopectin content may change the structure and chemical interaction forces of surimi protein, and these intermolecular forces may have a certain correlation with gel quality. Therefore, to verify this result, tapioca-starch-filled *N. virgatus* surimi gel was prepared. The physicochemical properties, microstructure, and molecular interaction of the complexed surimi gel were investigated. This study not only provides a reference for enhancing the quality of surimi products but also helps in understanding the underlying mechanisms of tapioca starch to improve the quality of surimi gel.

## 2. Materials and Methods

### 2.1. Materials

Commercially frozen *N. virgatus* surimi (AAA grade, 78.23% moisture content, 16.43% protein content) was provided by Yangjiang Yonghao Aquatic Products Co., Ltd. (Yangjiang, China). Native tapioca starch (food-grade; particle size of 19.30 μm; amylose content of 17%; amylopectin content of 83%; expansion capacity of 13.50–16.30 g/g; solubility of 3.40–95.90%) was purchased from Hebei Baiwei Biotechnology Co., Ltd. (Handan, China). All other chemicals were of analytical grade.

### 2.2. Preparation of Surimi Gel

Surimi gel was prepared based on a previous study [16] with some modifications. The surimi was minced in a Stephan chopper (UMC 5, Stephan Machinery Co., Hameln, Germany) at 900 rpm for 1 min. NaCl (2.5% (*w*/*w*)) was added to the surimi, chopped together for 1 min. Subsequently, the water amount was adjusted to 80%. Starch (0–14% (*w*/*w*)) was mixed into the surimi paste and minced at 1800 rpm for 3 min. The prepared surimi homogenate was filled into 25 mm plastic tubes and sealed at both ends. Two-stage heating gelation (heating at 40 °C for 30 min and heating at 90 °C for 20 min) was performed, and the heated surimi gel was placed at 4 °C for 12 h.

### 2.3. Determination of Gel Texture Properties

The surimi was left to equilibrate at room temperature and cut into cylinders (20 mm × 20 mm). The prepared samples were assessed using a texture analyzer (TA-XT plus C, Stab Micro System, Godalming, UK). The probe type for measuring the gel strength was a P/0.5 cylinder, and texture profile analysis (TPA) was performed using a P/0.5S spherical plunger [3]. The program was set as follows: the speed before the test was 5.0 mm/s, the speed during and after the measurement was 1.0 mm/s, the strain was 50%, and the trigger force was 5.0 g.

### 2.4. Determination of the Water-Holding Capacity (WHC)

Samples were sectioned to approximately 3 mm thickness, weighed accurately (*M*_1_), then folded and wrapped in two layers of paper and placed in centrifuge tubes for centrifugation (temperature: 4 °C, centrifugal force: 5000× *g*, time: 10 min). The weight (*M*_2_) after centrifugation was quickly recorded [17]. The calculation formula was as follows (Equation (1)):

(1) $$\mathrm{WHC}/\%=\frac{M_{2}}{M_{1}}\times 100$$

### 2.5. Determination of the Cooking Loss Rate (CLR)

The prepared gel was sliced and weighed accurately (*G*_1_) in a cooking bag (Jingsu Industries Ltd., Guangzhou, China). Subsequently, the samples were heated in hot water at 90 °C for 20 min. The samples were retrieved and placed at 4 °C for 24 h. The samples were wiped dry of surface moisture and weighed (*G*_2_) [16]. The calculation formula was as follows (Equation (2)):

(2) $$\mathrm{CLR}/\%=\frac{G_{1}-G_{2}}{G_{1}}\times 100$$

### 2.6. Determination of Whiteness

Gel samples were sliced into thin slices (5 × 20 × 20 mm^3^) and placed in clear plastic dishes [8]. The whiteness values were then measured using a colorimeter (3 nh, Shenzhen Technology Company, Shenzhen, China). The calculation was as follows (Equation (3)):

(3) $$\mathrm{Whiteness}=100-\sqrt{{\left(100-L*\right)}^{2}+a{*}^{2}+b{*}^{2}}$$

### 2.7. Determination of Low-Field Nuclear Magnetic Resonance (LF-NMR)

The moisture distribution of the sample was measured using a nuclear magnetic resonance imaging analyzer (NMI20-060H-I, Niumag Co., Suzhou, China). Using the CPMG pulse sequence, the relaxation time *T*_2_ of the sample was measured. The program was set as follows: magnet frequency SF = 21 MHz, spectral width SW = 200 MHz, recovery delay TW = 3000 ms, number of echoes NECH = 5000, and number of scans NS = 4. The *T*_2_ spectrum was obtained by means of cumulative sampling and inversion using Multi Exp Inv Analysis software (Version 2.0). The area of each peak in the integrated spectrum was accumulated, and the peak area indicated the percentage of water contained in the sample [18].

### 2.8. Light Microscopic Observation

The gel samples were cut into approximately 1.5 cm slices and placed into a solution containing sucrose at a concentration of 30% for dehydration (dehydration was considered complete when the samples floated to the surface). Afterward, the slices were immobilized in a mixture of 30% sucrose solution and optimal cutting temperature compound (OCT) in a 1:1 volume ratio for 4 h. Samples were fixed with OCT for an additional 4 h and placed in a −80 °C refrigerator to freeze for 1 h. Subsequently, the gel samples were cut into 30 μm slices with a freezing microtome [19], placed on glass slides, and stained with eosin for 2 min (the protein then appeared red). The morphological structure of the gel was observed using an inverted fluorescence inverted microscope (EVOS FL Auto2, USA Life Technologies Company, Carlsbad, CA, USA) at 200× magnification.

### 2.9. Scanning Electron Microscopy (SEM) Observation

The samples were sliced thinly, to about 1 mm, and fixed with glutaraldehyde solution (2.5%, *v*/*v*; pH 6.8) for 4 h. The samples were then rinsed twice with phosphate buffer (0.1 mol/L, pH 6.8), dehydrated with different gradient concentrations of ethanol, washed with absolute ethanol–tert-butanol at a ratio of 1:1, and finally washed with 100% tert-butanol. Surimi samples were freeze-dried for 48 h and examined via SEM (7610F, Japan Electronics Co., Ltd., Tokyo, Japan) at an accelerating voltage of 8 kV to observe the microstructure with a magnification of 15,000×.

### 2.10. Determination of Molecular Forces

Molecular forces determination was performed by mimicking the procedure used by Jiang [7]. Surimi samples (4 g) were added to 20 mL of 0.05 mol/L NaCl solution (S1), 0.6 mol/L NaCl solution (S2), 1.5 mol/L urea + 0.6 mol/L NaCl solution (S3), 8 mol/L urea + 0.6 mol/L NaCl solution (S4), and 0.6 mol/L NaCl + 8 mol/L urea + 0.5 mol/L β-mercaptoethanol (S5). The mixture was homogenized using a high-speed disperser (IKA T-18 homogenous disperser, Southeast Scientific Instruments Co., Ltd. Guangdong, China) for 5 min and left to stand for 1 h at 4 °C. Subsequently, the mixture was centrifuged at 8000 r/min for 15 min in a freezing centrifuge (JIDI-21K, Guangzhou Vicki Technology Co., Ltd., Guangzhou, China). The protein content of the supernatant was measured following the procedure used by Lowry [10]. The formula for calculating non-covalent bonds is as follows (Equations (4)–(7)):

(4) Ionic bonds=c(S2−S1)

(5) $$Hydrogen\;bonds=c\left(S3-S2\right)$$

(6) Hydrophobic interactions=c(S4−S3)

(7) Disulfide bonds=c(S5−S4)

### 2.11. Fourier Transform Infrared (FT-IR) Spectroscopy

The freeze-dried samples were mixed with potassium bromide (KBr) in a mass ratio of 1:100, ground into powder in an agate dish, and compressed into tablets. FT-IR spectroscopy (VERTEX70, Bruker Optice Inc., Carlsruhe Karlsruhe, Germany) was performed by scanning the sample 32 times in the wave number range of 4000~500 cm^−1^. The protein secondary structure was derived by fitting using Peakfit 4.12 software.

### 2.12. Statistical Analysis

Data are presented as the mean ± standard deviation of results from five experiments. The plotting software used was Origin 2023, and in all data analysis by means of one-way analysis of variance (ANOVA) and Duncan’s test (SPSS 22.0), a value of *p* < 0.05 was considered to indicate a statistically significant difference.

## 3. Results

### 3.1. Effect of Starch on the Physicochemical Properties of Surimi Gel

#### 3.1.1. Texture Profile Analysis

The gel strength and TPA value are some of the indicators used to measure the quality of surimi products. Proteins and starches aggregate and gel through heating, thereby promoting the textural properties of food [20]. The textural properties of *N. virgatus* surimi gels at different levels of tapioca starch addition (0–14% (*w*/*w*)) are shown in Table 1. Surimi gel containing tapioca starch showed significantly improved gel strength, hardness, gumminess, and chewiness (*p* < 0.05) but slightly decreased cohesiveness (*p* < 0.05). When starch absorbs water and expands at a specific gelatinization temperature, the starch particles squeeze and fill voids in the gel matrix, which leads to the increased hardness and gel strength of the surimi gel [5]. The gel strength, hardness, springiness, and gumminess of the gels were enhanced to varying degrees as the tapioca starch content increased, achieving maximum values with the addition of 12% tapioca starch (*p* < 0.05). This is because the increase in the amount of added tapioca starch led to an increase in tapioca starch molecules per unit volume and an increase in the content of hydrogen bonds between molecules, bringing about a firm and dense network structure [9]. Furthermore, the gel texture did not change when 14% tapioca starch was added. This is because too much starch distributed within the protein structure can lead to the saturated crosslinking of polysaccharides with proteins and hinder protein–protein interactions [7,21]. Eventually, gel properties no longer change or show a downward trend. The data are similar to those from other studies [8] suggesting that excess starch decreases the gel properties of myofibrillar protein–starch complexes.

#### 3.1.2. WHC and CLR

**Figure 1:**
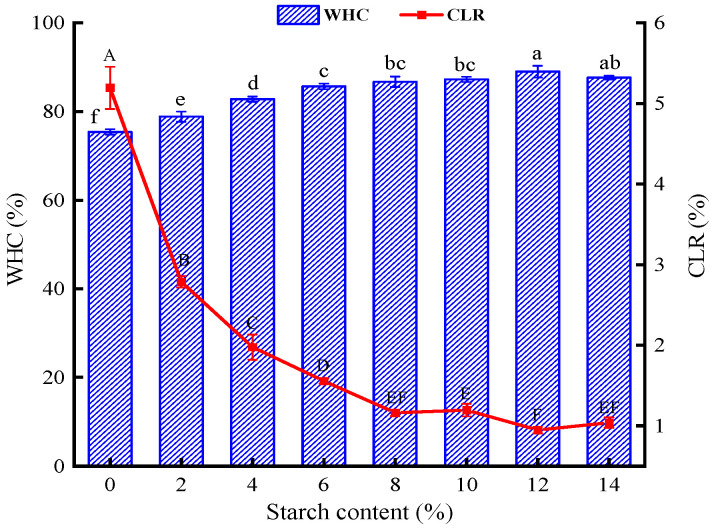
Effect of different contents of tapioca starch on the WHC and CLR of surimi in the surimi gel. The data with an identical letter indicate no significant difference between them (*p* > 0.05).

In general, a higher WHC and a lower CLR mean that the surimi gel can lock in plenty of water, forming a dense network structure [22]. The WHC values of the surimi gels with the addition of tapioca starch were all enhanced and the CLR values were all decreased in comparison to those for the surimi gels without tapioca starch (Figure 1). Moreover, the WHC maintained an upward trend with increasing tapioca starch addition (*p* < 0.05), except for the 14% group. The addition of 12% tapioca starch increased the WHC from 75.40 (in the control group) to 89.01%. As the heat-treated starch granules absorb water around the protein, the swollen starch granules squeeze the gel matrix, helping the surimi gel to retain moisture [9]. This is especially the case for starches with a high amylopectin content. When the starch granular structure is damaged, amylopectin undergoes cleavage, and more hydroxyl groups are then exposed to water [23]. This results in an increase in the WHC of the gel. Conversely, when the amount of added tapioca starch reached 14%, the WHC did not continue to rise, and the CLR did not continue to fall. Luo et al. [5] came to the same conclusion, finding that a certain amount of starch can bind more water, increasing the WHC value to a certain extent. However, excess starch competes with myofibrillar protein for water, preventing the dehydrated starch from fully expanding.

#### 3.1.3. Water Distribution

**Figure 2:**
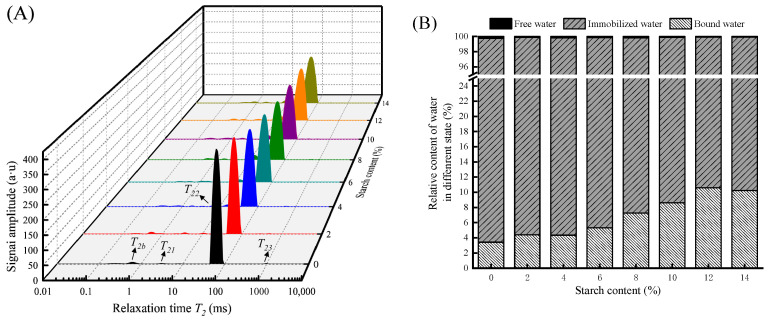
Effect of different contents of tapioca starch on the spin-spin relaxation times of surimi gel (**A**). Relative area percentages of the *T*_21_, *T*_22_, and *T*_23_ peaks (**B**).

LF-NMR is a novel method for assessing the distribution of moisture states in food [5]. Figure 2A mainly reflects the influence of tapioca starch addition on the transverse relaxation time spectra of the gel. Three peaks in three regions were detected, namely *T*_21_ (1–10 ms), *T*_22_ (10–100 ms), and *T*_23_ (>100 ms), representing bound water, immobilized water, and free water, respectively [24]. In general, immobilized water dominates the water content in the surimi gels and is inseparable from the gel network structure [25].

The relaxation times of each peak in the integral spectrum were calculated and are presented in Table 2. The *T*_22_ of immobilized water gradually decreased with an increasing content of tapioca starch (*p* < 0.05), which indicated that tapioca starch raised the binding of hydrogen protons and lowered the free water. The higher the addition amount, the more hydrogen bonds were generated to bind tightly with the protein, thus reducing the mobility of the water [3]. Moreover, the starch absorbs some of the water molecules in the surimi gel, causing more water to be locked by starch and adsorbed on the gel [5], improving the WHC of the surimi gel. The relaxation time of immobilized water (*T*_22_) was minimized with 12% tapioca starch, reflecting the strongest binding ability to water and having the best WHC.

The relative contents of water in different states in the surimi gel are shown in Figure 2B. The immobilized water content accounted for more than 89%, and the other two types of water content accounted for less. With the addition of tapioca starch, the higher the starch content, the more the bound water content increased and the free water content decreased. The gels had the best ability to bind water molecules when the starch addition amount was 12%. On the one hand, a small amount of free water in the gel is converted into bound water, stabilizing the water molecules in the gel network [25]. On the other hand, starch contains numerous hydroxyl groups, which helps to bind more water [7]. Therefore, starch fills the gel network to some extent and could reduce the gel pore size, preventing water from leaking out of the gel pores.

#### 3.1.4. Whiteness

As shown in Table 3, after the addition of tapioca starch, the L*, a*, and b* values of the surimi gel decreased (*p* < 0.05), indicating the decreased whiteness of the surimi gel (*p* < 0.05). In addition, the higher the amount of starch added, the more adverse the effect on the whiteness. According to scholars, the whiteness of surimi gel is related to the structure of the gel, as well as the type and color of additives [7]. Consequently, tapioca starch is not conducive to the production of whiteness. The same conclusion was obtained by Luo et al. [5] in hairtail surimi gels with potato starch. Large-size starch granules have a strong swelling capacity when heated and can fill the voids in the gel network structure. This leads to decreased voids and to light being unable to pass through the gel surface, thereby reducing whiteness [5]. However, the results of this study differ from existing reports in which starch was found to increase the whiteness value of surimi gel [8]. This discrepancy may be due to differences in the interactions between different starch types and surimi gel.

### 3.2. Effect of Starch on the Microstructure of Surimi Gel

#### 3.2.1. Light Microscopic Observation

**Figure 3:**
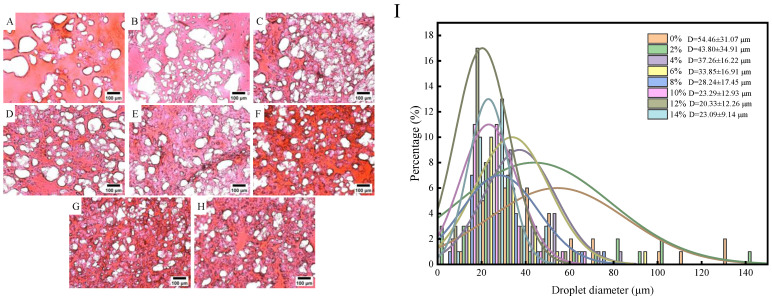
Effect of different contents of tapioca starch on the light microscopic picture (×200) and pore size distribution of surimi gels. (**A**): control; (**B**–**H**): the surimi gel containing tapioca starch in concentrations of 2, 4, 6, 8, 10, 12, and 14 per 100 g of surimi, respectively; (**I**): Average cavities of surimi gel with different contents of tapioca starch.

Figure 3 present the results of light microscopy, which mainly reflect the dispersion and morphology of tapioca starch within the gel structure. Figure 3A intuitively shows that the morphological structure of the surimi gels without tapioca starch was relatively diffused, with an average cavity size of 54.46 μm (Figure 3I). However, the surimi gel exhibited a more regular structure and the space occupied by cavities gradually decreased with an increasing content of tapioca starch. The average cavity size of the surface decreased to the range of 20.33–43.80 μm (Figure 3I). It is worth mentioning that the morphological structure became denser when the starch content was 12%, and the average cavity size decreased to 20.33 μm (Figure 3I). Our result is compatible with those of other studies showing that starch can fill the network structure of myofibrillar proteins and reduce the average cavity size [9]. The reduced cavity size of gels can be explained by the tapioca starch granules being heated and expanding to form web-like fibers within the cavities, acting as bridges to hold the proteins together [26]. However, different starch varieties have different particle sizes [13]. Tapioca starch, possessing large particle sizes, easily absorbs water and expands, and it has a better filling effect on the gel network structure. In addition, the powerful dehydrating effect of tapioca starch can result in the absorption of water within the myofibrillar protein. Thus, dehydrated myofibrillar proteins have the potential to provide a better hydrophobic environment that promotes the unfolding of protein molecules and exposes hydrophobic groups during heat treatment [27]. The highly compact network structure prevents the flow of water, which has a positive effect on maintaining moisture in the gel [5]. Similarly, the gel structure did not change much when tapioca starch was added to 14%, further indicating that there is a threshold for the crosslinking of myofibrillar protein with tapioca starch. This phenomenon aligns with the conclusion from the water-holding studies.

#### 3.2.2. SEM Observation

**Figure 4:**
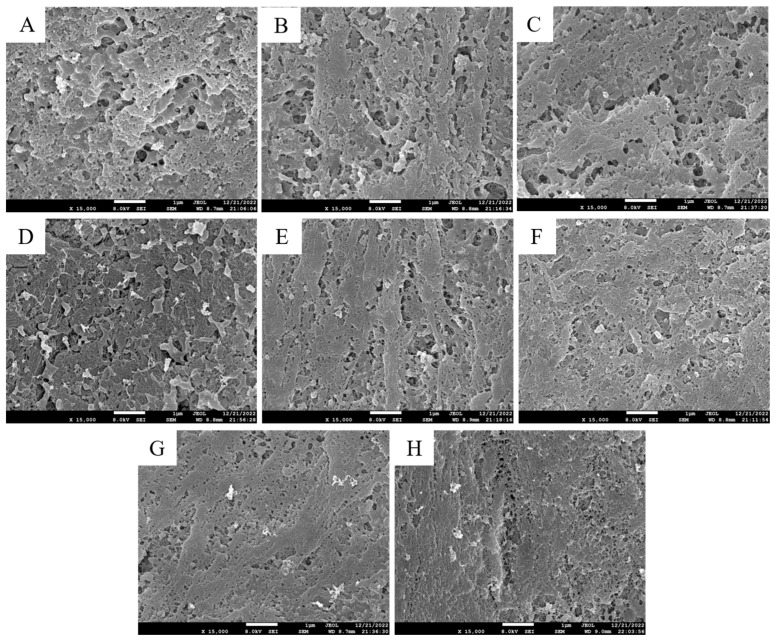
Effects of different contents of tapioca starch on the scanning electron microscope photograph (×15,000) of the surimi gel. (**A**): control; (**B**–**H**): the surimi gel containing tapioca starch in the concentrations of 2, 4, 6, 8, 10, 12, and 14 per 100 g of surimi, respectively.

SEM is a more intuitive analysis approach that can reflect the differences in the microstructure of surimi gels. Figure 4A clearly shows that the surimi gel without tapioca starch had an extremely rough and loose surface, but surimi gels containing tapioca starch exhibited a low pore count and narrow pores. When the tapioca starch addition reached 12%, the pores were minimal, and the gels showed a compact and uniform network microstructure. As a hydrophilic macromolecule, tapioca starch could serve as a hydrophilic filler for the network structure of surimi gels, enhancing the physical stability of surimi gels by locking free water and interconnected water channels [28]. In particular, tapioca starch increased the amount of water or some dry substances retained, thereby altering the microstructure [9]. As a result, tapioca starch could stabilize water molecules in the gel network structure and induce the formation of a compact and uniform network structure. This effect was enhanced with an increasing tapioca starch addition amount. Wu et al. [29] reached the same conclusion that amylopectins caused *scomberomorus phonics* surimi gels to form a firm and ordered three-dimensional gel network with improved WHC. However, the gel structure did not change much at 14% starch addition. Excessive starch competition for water in proteins can lead to protein dehydration wherein proteins cannot fully unfold, resulting in some proteins not reaggregating and hindering the formation of gel network structures. The SEM observations were the same as those described above for WHC and CLR. A dense network structure helps the gel to intercept water molecules and improve the WHC, while a loose network structure is not conducive to a reduction in the gel’s CLR value. Consequently, when the tapioca starch addition amount reached 12%, the surimi gel exhibited a stable and ordered three-dimensional gel network.

### 3.3. Analysis of the Molecular Interactions

#### 3.3.1. Chemical Interaction Forces

**Figure 5:**
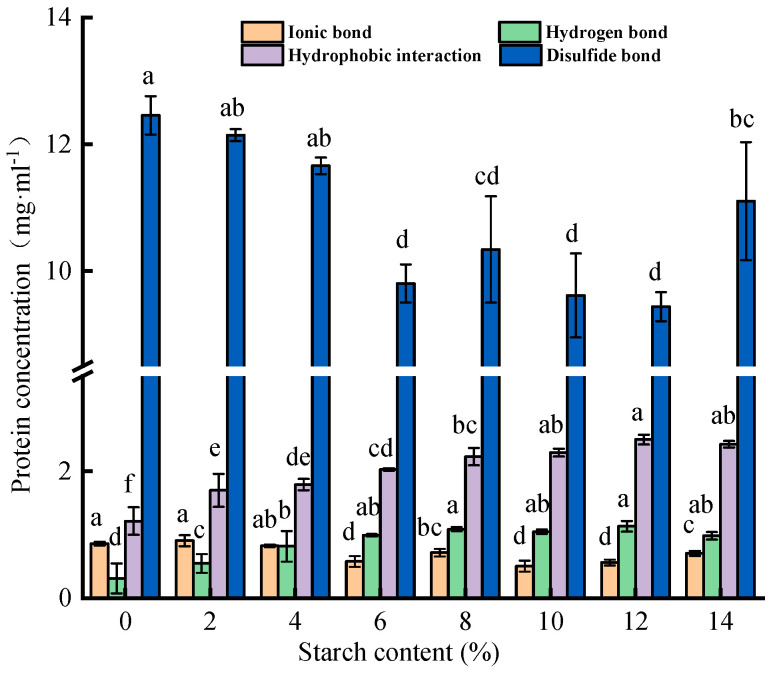
Effect of different contents of tapioca starch on the chemical force of the surimi gel. The data with an identical letter indicate no significant difference between them (*p* > 0.05).

In general, heat exposure induces the opening of myofibrillar proteins’ helical structures, with more sulfhydryl groups and hydrophobic amino acids exposed, thereby producing covalent crosslinking of disulfide bonds and hydrophobic interactions [30]. Figure 5 shows that the disulfide bonds and hydrophobic interactions contributed more to surimi gel matrix maintenance, while ionic and hydrogen bonds contributed less. Tapioca starch decreased the disulfide and ionic bonds but promoted hydrophobic interactions and hydrogen bond formation (*p* < 0.05). Tapioca starch causes a loss of thiol group content by releasing hydroxyl groups and reacting with thiol groups, hindering the generation of disulfide bonds [31]. Therefore, with the addition of tapioca starch, disulfide bonds showed a downward trend (*p* < 0.05). This conclusion is similar to a previous finding that the potato-starch-induced reduction in disulfide bonds may be influenced by the starch granular structure [10]. This change may be because, during the heating process, tapioca starch binds to the thiol groups on myofibrillar proteins and undergoes a non-disulfide bonding polymerization reaction [32].

More importantly, exposure to hydrophobic sites in surimi proteins can enhance hydrophobic interactions, allowing proteins to aggregate with each other and thus induce gel network formation, which is significant for stabilizing the surimi matrix [4,10]. Figure 5 intuitively shows that the hydrophobic interactions continued to rise with an increasing content of tapioca starch, reaching a maximum value for the surimi gel with 12% added tapioca starch (from 1.21 mg/mL to 2.50 mg/mL). But when the content of tapioca starch increased to 14%, hydrophobic interactions in the surimi gel began to decline. On the one hand, the increase in tapioca starch results in a gradual increase in water absorption, and the water molecules in the surimi gel can be retained within a certain range, thus increasing hydrophobic interactions. However, excessive water absorption prevents the dispersion or dissolution of the protein itself in water, which affects the hydrophobic interactions in the system to a certain extent [33]. On the other hand, the hydroxyl groups in starch cross-connect with hydrophobic sites or other residues in myofibrillar protein molecules as fillers, increasing hydrophobic interactions [4]. In addition, the amylopectin in tapioca starch can prevent the excessive unfolding of proteins and prevent the aggregation of hydrophobic groups that lead to partial shielding of hydrophobic regions, thus increasing the surface hydrophobicity [31]. The increase in surface hydrophobicity has a positive effect on the formation of hydrophobic interactions, prompting the gel to form a dense network structure and stronger WHC.

As shown in Figure 5, tapioca starch enhanced the hydrogen bonds in surimi gel to a certain extent (*p* < 0.05). As we know, starch forms many hydrogen bonds during regeneration [34]. However, hydrogen bonds play a major role in maintaining the steadiness of bound water and increasing the rigidity of surimi gels. Therefore, the formation of hydrogen bonds improved the textural properties of the surimi gel (Table 1). In addition, tapioca starch weakens the production of ionic bonds. This may also be because starch hinders the formation of ionic bonds in the protein. Generally, tapioca starch addition was beneficial to increasing the hydrogen bonds and hydrophobic interactions (*p* < 0.05), which enhanced the quality and stabilized the network structure of the surimi gels.

#### 3.3.2. FT-IR Spectroscopy

**Figure 6:**
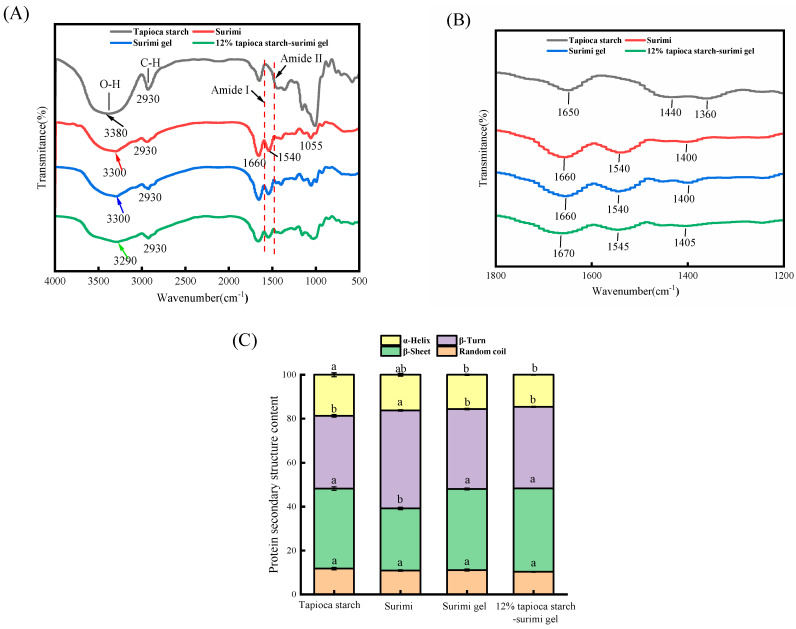
FT-IR spectral of tapioca starch, surimi, surimi gel, and tapioca starch–surimi gel (tapioca starch content of 12%) (**A**,**B**), and the change in secondary structure (**C**). The data with an identical letter indicate no significant difference between them (*p* > 0.05).

Molecular interactions and structural changes in surimi gels were explored in depth using FT-IR. Figure 6A shows the FT-IR (4000–500 cm^−1^) spectra of tapioca starch, surimi, surimi gel, and surimi gel supplemented with 12% tapioca starch. The 3500–3000 cm^−1^ range is known as the amide A band, representing the stretching and vibrations of O-H and N-H, which are closely related to hydrogen bonds and hydrophobic bonds [35]. The amide A band can also be used to determine the interaction between proteins and water molecules [30]. The O-H elongation band peaks of tapioca starch, surimi, surimi gel, and surimi gel with 12% tapioca starch were located at 3380, 3300, 3300, and 3290 cm^−1^, respectively. These data indicate that starch addition caused the peak for surimi gel to move towards a lower wavenumber. A lower wavenumber is beneficial for the formation of intramolecular hydrogen bonds by O-H, which are closely linked to the binding ability of water molecules [10]. Therefore, starch can change the water binding capacity of surimi gels. This conclusion echoes the increase in bound water in the surimi gel, as confirmed by the LF-NMR assay. The reason for this change may be that the environment in the vicinity of the aliphatic O-H and N-H groups is altered, which enhances the stretching and bending vibrations of O-H and N-H. This change is related to hydrophobic interactions [36]. At the same time, starch heating exposes the O-H group in the glucose molecule, which may also cause changes in hydrogen bonds [37].

Figure 6B shows the amide I band in the range of 1600–1700 cm^−1^, and the percentage content of the protein secondary structure was plotted by means of Peakfit 4.12 fitting (Figure 6C). The peak bands of tapioca starch and surimi were located at wavenumbers of 1650 cm^−1^ and 1660 cm^−1^, respectively. The peaks for surimi gel and the surimi gel with 12% tapioca starch were located at wavenumbers of 1660 cm^−1^ and 1670 cm^−1^, respectively, indicating that the addition of starch into the surimi gel prompted the variation in the α-helical structure into β-turns and β-sheets. Compared to those of the surimi gel, the α-helix content of the surimi gel with 12% tapioca starch decreased from 15.62% to 14.61% (*p* > 0.05), while the relative β-turn and β-sheet contents increased from 36.33% to 37.05% and from 36.94% to 37.99% (*p* > 0.05), respectively (Figure 6C). Thus, starch molecules induced the unfolding of the myofibrillar protein, enhanced hydrogen bonds and hydrophobic interactions, and contributed to shifts from α-helices to β-turns and β-sheets in the surimi gel [38]. This finally led to a red shift of the peak of the amide I band in the surimi gel. It has been proved that the gel strength of myofibrillar protein is positively correlated to the amount of β-sheets, β-turns, and random coils but is negatively correlated to the amount of α-helices [39]. The cause of this change should be the interaction between myofibrillar protein molecules. Increased β-sheet and β-turn contents also indicate an orderly change in the protein network structure [38]. The transformation of the protein structure in the surimi gel was consistent with the observed changes in the surimi gel microstructure.

### 3.4. Correlation Analysis

**Figure 7:**
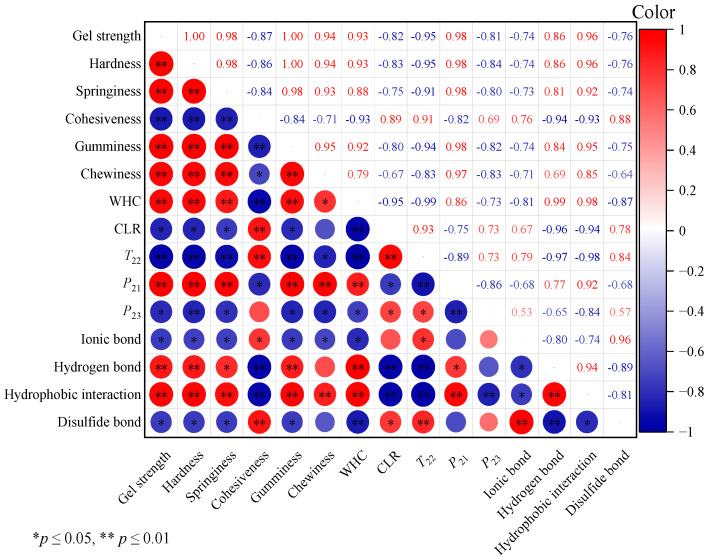
Correlation analysis between the gel strength of surimi gel and other indexes. (The red number and circle represent a positive correlation, and the blue number and circle represent a negative correlation. The closer the correlation coefficient is to 1.00, the more significant the positive correlation between the two is, and vice versa).

The relationships between the gel texture properties, moisture distribution status, and tapioca starch–surimi gel molecular interaction forces were further elucidated by correlation analysis. As shown in Figure 7, the correlation index value between the gel strength, hardness, springiness, gumminess, and chewiness of the surimi gel was close to 1.00, indicating a significant positive correlation between them (*p* < 0.01). At the same time, in terms of the moisture distribution status, the correlation index values between gel strength, hardness, springiness, gumminess, and chewiness and the WHC and bound water were 0.93 and 0.98, respectively, reflecting significant positive correlations (*p* < 0.01). However, it was negatively correlated with CLR, free water, and relaxation time (*T*_22_) (*p* < 0.05). It was thus further proved that tapioca starch can reduce the free water in surimi gel, and water molecules can be stabilized in the gel matrix to form a compact gel network structure, which helps to improve the gel quality. In addition, the gel strength, WHC, and springiness were positively correlated with hydrogen bonds and hydrophobic interactions (R = 0.90, *p* < 0.01) and negatively correlated with ionic bonds and disulfide bonds (*p* < 0.05). It can be concluded that the change in textural properties in the surimi gel was related to molecular interaction forces, and the increased hydrogen bonds and hydrophobic interactions were more favorable to improving the quality of the surimi gel.

## 4. Conclusions

Tapioca starch significantly improved the quality and microstructure of surimi gel. This is mainly due to the superior water absorption, swelling, and filling effects of tapioca starch. The results of chemical force and FT-IR analysis also confirmed the above conclusions. Tapioca starch enhanced the hydrogen bonds and hydrophobic interactions in the surimi gel system and promoted a conformational transition of the secondary structure of the surimi gel protein from α-helices to β-sheets and β-turns. This is due to the binding of some hydroxyl groups in the tapioca starch to myofibrillar protein residues, resulting in a compact and ordered three-dimensional gel network structure. The excellent gel network structure endowed the surimi gels with enhanced WHC and texture characteristics. In addition, surimi gels containing 12% starch exhibited excellent texture values and water retention, while an excessive starch concentration (14%) disrupted the integrity of the gel matrix. Therefore, tapioca starch filling is a useful strategy to optimize the gel quality of surimi gel. The results of this research will also provide valuable information for subsequent studies of the interaction mechanism between polysaccharides and proteins.

## Figures and Tables

**Table 1:** Effects of different contents of tapioca starch on texture of the surimi gel.

Tapioca Starch (g per 100 g)	Gel Strength/N	Hardness/N	Springiness	Cohesiveness	Gumminess	Chewiness
0	7.59 ± 0.02 ^g^	12.61 ± 0.58 ^g^	0.77 ± 0.00 ^c^	0.66 ± 0.00 ^a^	8.64 ± 0.11 ^f^	6.66 ± 0.08 ^d^
2	8.51 ± 0.11 ^f^	13.95 ± 0.21 ^f^	0.78 ± 0.00 ^c^	0.65 ± 0.00 ^ab^	9.07 ± 0.43 ^f^	6.99 ± 0.42 ^d^
4	10.13 ± 0.22 ^e^	15.48 ± 0.15 ^e^	0.77 ± 0.01 ^c^	0.65 ± 0.01 ^abc^	10.19 ± 0.09 ^e^	7.90 ± 0.17 ^d^
6	11.75 ± 0.19 ^d^	17.64 ± 0.36 ^d^	1.01 ± 0.34 ^bc^	0.65 ± 0.00 ^bcd^	11.47 ± 0.41 ^d^	9.97 ± 0.69 ^c^
8	14.06 ± 0.20 ^c^	19.69 ± 0.69 ^c^	1.20 ± 0.33 ^ab^	0.64 ± 0.00 ^d^	12.65 ± 0.45 ^c^	10.77 ± 0.38 ^c^
10	15.17 ± 0.31 ^b^	20.69 ± 0.29 ^b^	1.21 ± 0.32 ^ab^	0.64 ± 0.01 ^cd^	13.53 ± 0.22 ^b^	18.79 ± 0.40 ^b^
12	17.48 ± 0.23 ^a^	24.00 ± 0.55 ^a^	1.41 ± 0.01 ^a^	0.64 ± 0.00 ^d^	15.42 ± 0.33 ^a^	21.36 ± 1.13 ^a^
14	17.27 ± 0.10 ^a^	23.47 ± 0.03 ^a^	1.40 ± 0.00 ^ab^	0.65 ± 0.00 ^bcd^	15.26 ± 0.09 ^a^	21.54 ± 0.23 ^a^

Note: The data are expressed in the form of the mean ± standard deviations (*n* = 5). Different letters within the same row indicate significant differences between mean values (*p* < 0.05).

**Table 2:** Effects of different contents of tapioca starch on relaxation time (*T*_2_) of the surimi gel.

Tapioca Starch (g per 100 g)	*T*_2*B*_	*T*_22_	*T*_23_
0	2.36 ± 0.24 ^ab^	44.49 ± 0.00 ^a^	784.70 ± 31.81 ^a^
2	2.97 ± 0.21 ^a^	41.50 ± 0.00 ^b^	309.09 ± 73.66 ^c^
4	2.12 ± 0.64 ^bc^	36.12 ± 0.00 ^c^	459.16 ± 16.99 ^b^
6	1.48 ± 0.10 ^c^	33.70 ± 0.00 ^d^	472.95 ± 65.86 ^b^
8	1.66 ± 0.65 ^bc^	31.44 ± 0.00 ^e^	483.43 ± 37.82 ^b^
10	1.64 ± 0.06 ^bc^	30.74 ± 1.22 ^e^	322.37 ± 18.63 ^c^
12	1.82 ± 0.43 ^bc^	28.67 ± 1.13 ^f^	460.15 ± 39.17 ^b^
14	1.81 ± 0.39 ^bc^	29.33 ± 0.00 ^f^	465.42 ± 43.12 ^b^

Note: Different letters within the same row indicate significant differences between mean values (*p* < 0.05).

**Table 3:** Effects of different contents of tapioca starch addition on the whiteness of the surimi gel.

Tapioca Starch (g per 100 g)	L*	a*	b*	Whiteness
0	71.68 ± 0.17 ^a^	−2.51 ± 0.03 ^a^	5.17 ± 0.19 ^a^	71.10 ± 0.20 ^a^
2	70.21 ± 0.33 ^b^	−2.73 ± 0.02 ^bc^	4.24 ± 0.11 ^b^	69.78 ± 0.34 ^b^
4	68.57 ± 0.11 ^c^	−2.81 ± 0.05 ^cd^	4.04 ± 0.18 ^bc^	68.18 ± 0.12 ^c^
6	66.59 ± 0.06 ^d^	−2.85 ± 0.05 ^d^	3.79 ± 0.14 ^cd^	66.25 ± 0.06 ^d^
8	65.31 ± 0.06 ^e^	−2.83 ± 0.06 ^d^	3.49 ± 0.14 ^e^	65.01 ± 0.05 ^e^
10	64.01 ± 0.07 ^f^	−2.79 ± 0.05 ^cd^	3.38 ± 0.03 ^e^	63.74 ± 0.06 ^f^
12	62.59 ± 0.45 ^g^	−2.81 ± 0.01 ^cd^	3.53 ± 0.12 ^de^	62.32 ± 0.44 ^g^
14	62.00 ± 0.16 ^h^	−2.66 ± 0.06 ^b^	3.48 ± 0.27 ^e^	61.75 ± 0.18 ^h^

Note: Different letters within the same row indicate significant differences between mean values (*p* < 0.05).

## Data Availability

Data are contained within the article.
